# Protectin DX increases survival in a mouse model of sepsis by ameliorating inflammation and modulating macrophage phenotype

**DOI:** 10.1038/s41598-017-00103-0

**Published:** 2017-03-07

**Authors:** Haifa Xia, Lin Chen, Hong Liu, Zhipeng Sun, Wen Yang, Yiyi Yang, Shunan Cui, Shengnan Li, Yaxin Wang, Limin Song, Amro Fayez Abdelgawad, You Shang, Shanglong Yao

**Affiliations:** 1 Department of Anesthesiology, Union Hospital, Tongji Medical College, Huazhong University of Science and Technology, Wuhan, 430022 China; 2 Department of Critical Care Medicine, Union Hospital, Tongji Medical College, Huazhong University of Science and Technology, Wuhan, 430022 China; 3 Institute of Anesthesiology and Critical Care Medicine, Union Hospital, Tongji Medical College, Huazhong University of Science and Technology, Wuhan, 430022 China; 4 Faculty of Medicine, Benha University, Benha, 002013 Egypt

## Abstract

Recently, a serial of studies have demonstrated that lipid mediators derived from Omega-3 fatty acid docosahexaenoic acid have pro-resolving or anti-inflammatory effects in many inflammatory diseases. Here, we sought to evaluate whether Protectin DX (PDX, an isomer of Protecin D1), a newly identified lipid mediator, could protect mice against sepsis and explore the underling mechanism. Animal model of sepsis was established by cecum ligation and puncture (CLP). We found that PDX increased overall survival rate within eight days and attenuated multiple organ injury in septic mice. In addition, PDX reduced pro-inflammatory cytokines and bacterial load 24 h after CLP. Moreover, PDX promoted phagocytosis of peritoneal macrophages and increased the percentage of M2 macrophages in peritoneum of septic mice. *In vitro*, M2 macrophage markers (Arg1 and Ym1) and its transcriptional regulator (peroxisome proliferator-activated receptor-γ, PPAR-γ) were upregulated in Raw264.7 macrophages challenged with PDX. GW9662 (a PPAR-γ inhibitor) and PPAR-γ siRNA abrogated the induction of Arg1 and Ym1 by PDX in Raw264.7 cells. Taken together, our results suggest that PDX is able to promote M2 polarization, enhance phagocytosis activity of macrophage and accelerate resolution of inflammation, finally leading to increased survival rate of septic mice.

## Introduction

Sepsis is a leading cause of death in critical ill patients worldwide^1^. Approximately 40–70% of the mortality is attributed to severe sepsis and sepsis induced multiple organ failure^2^. Uncontrolled inflammation induced by pathogens and subsequent immune disorder or immunosuppression is an underlying mechanism of sepsis^3^. It is well-recognized that the resolution of acute inflammation is an active process. A growing body of evidences indicated that derivants of docosahexaenoic acid (DHA), such as protectins, maresins and resolvins, play an important role in regulating the inflammatory response and orchestrating the host response to injury as well as infection^4–7^.

PDX (10S, 17S-dihydroxy-docosa-4Z, 7Z, 11E, 13Z, 15E, 19Z-hexaenoic acid) is a new lipid mediator derived from DHA by double lipoxygenation and is an isomer of protectin D1 (PD1, 10R, 17S-dihydroxydocosa-4Z, 7Z, 11E, 13E, 15Z, 19Z-hexaenoic acid), both of them can be found in DHA challenged neutrophils or inflammatory exudates^8, 9^. PDX not only displayed the classic function of protectins in the process of inflammation resolution but also exhibited anti-aggregatory property in human neutrophil^10–12^. However, its role in sepsis remains elusive.

Macrophages are key component in innate immunity, homeostasis, and other inflammatory diseases. They adopt two distinct phenotypes following activation, the classically activated macrophages (termed as M1 phenotype) or alternatively activated macrophages (termed as M2 phenotype), depending on the surrounding environments^13, 14^. M1 macrophages are characterized by producing pro-inflammatory cytokines (IL-1β, IL-6, TNF-α, etc.), while M2 phenotype participate in anti-inflammatory cytokines (IL-4 and IL-10) secretion and tissue repair^15, 16^. Prime macrophages towards M2 polarization could be an active process which is essential for sepsis treatment^17^. Recent studies had shown that Resolvin D1, Merasin1 and its precursor DHA have great potential in modulating the phenotype of macrophage^18, 19^. Furthermore, as an important transcriptional regulator of macrophage and adipocyte differentiation, PPAR-γ was found to be upregulated in PDX-treated tissue and cell^20, 21^. Therefore, we hypothesize that PDX can protect mice against sepsis by modulating macrophage differentiation and facilitating the resolution of inflammation.

In the present study, our data demonstrate that administration of PDX increases the overall survival rate of septic mouse via promoting the resolution of inflammation and bacterial clearance, regulating macrophage polarization. PDX might be a novel therapeutic approach to resolve sepsis in the future.

## Results

### PDX reduced mortality of mice after CLP

**Figure 1:**
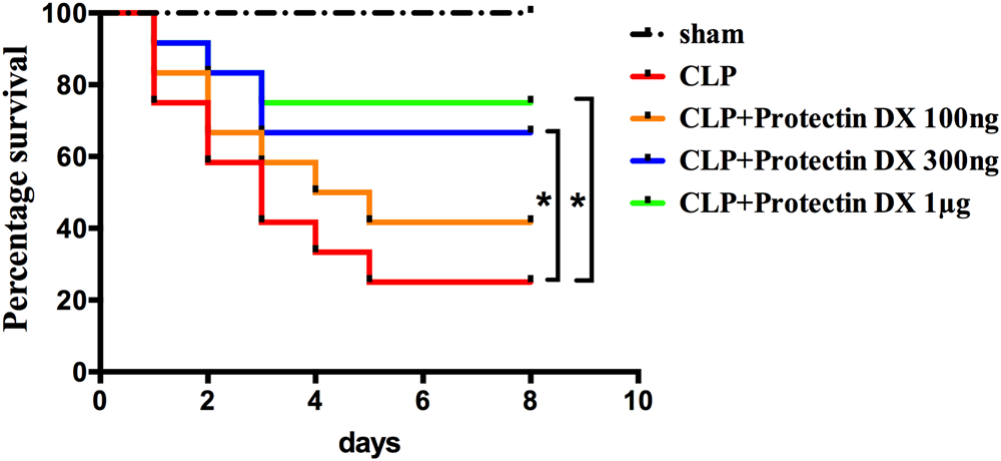
PDX reduced mortality of mice after CLP. Kaplan–Meier survival curves were used to estimate survival rate in five groups. Survival rate differences were analyzed by the log-rank test. An equal volume of vehicle was given in both the sham and CLP groups, mice treated with PDX (300 ng or 1000 ng) 1 h after CLP showed higher survival rate as compared with the CLP group (n = 12). *P < 0.05.

In order to determine the most effective dose of PDX based on an animal model of sepsis, mice were closely observed for 8 days after CLP. All mice survived within 8 days in sham group. As shown in Fig. 1, the mortality rate in CLP group was extremely high and 100 ng PDX did not influence the survival rate in septic mice. In contrast, mice treated with PDX (300 ng or 1000 ng) after surgery displayed higher survival rate as compared with CLP group. However, no significant difference was observed between PDX 300 ng and PDX 1000 ng regarding to survival rate. Hence, PDX at 300 ng was chosen for the subsequent analyses.

### PDX prevented multiple-organ injury in sepsis

**Figure 2:**
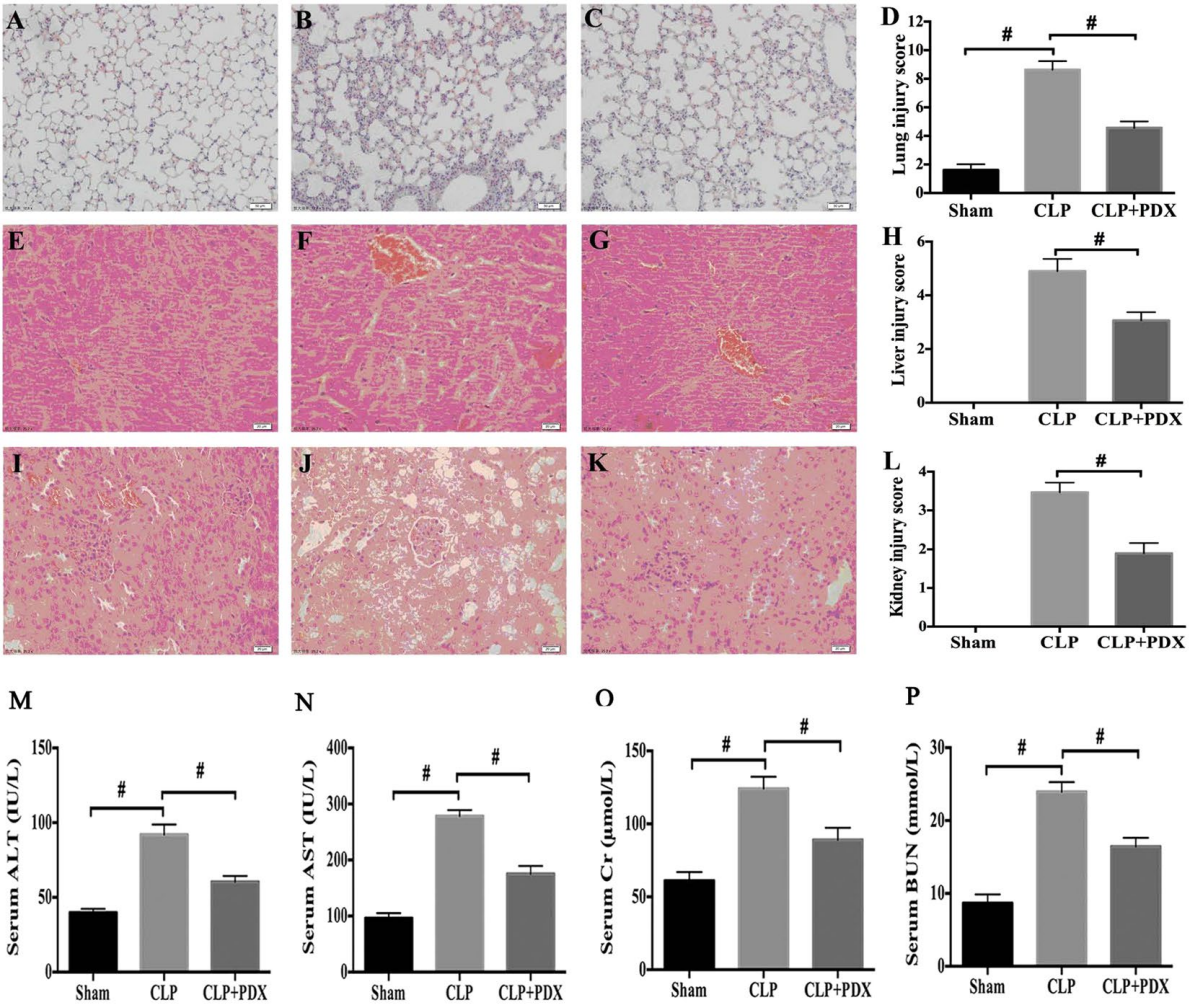
PDX protected CLP mice from multiple-organ injury. Representative images of lung (magnification 200X), liver and kidney (magnification 400X) histology staining with hematoxylin and eosin. The normal lung (**A**), liver (**E**) and kidney (**I**) structure in sham group. CLP mice showed that lung (**B**), liver (**F**) and kidney (**J**) were all injured. PDX mitigated organ injury in septic mice (**C**,**G** and **K**). Organ injury scores in the three groups (**D**,**H** and **L**). The concentration of alanine aminotranferase (ALT) (**M**), aspartate amniotransferase (AST) (**N**), creatinine (Cr) (**O**) and blood urea nitrogen (BUN) (**P**) in blood was determined by using an automated chemistry analyzer. Data are presented as means ± SEM (n = 6). ^#^ *P* < 0.01.

Mice in the sham group displayed normal tissue histology (Fig. 2A,E,I). Major organs suffer from sepsis in mice after CLP, for instance, lung tissues were characterized by neutrophil infiltration, hemorrhage, alveolar disarray, and hyaline membrane (Fig. 2B), the liver exhibited increased parenchyma inflammation and degenerative changes (Fig. 2F), whilst the kidney showed increased necrosis and capsular inflammation (Fig. 2J). However, administration of PDX attenuated the organ injury (Fig. 2C,G,K). And these results are in consistent with organ injury score (Fig. 2D,H,L). In addition, blood biochemistry for liver and kidney injury were also measured. The normal function of liver was damaged following CLP as alanine aminotranferase (ALT) and aspartate amniotransferase (AST) increased significantly (Fig. 2M and N), renal function was also compromised in septic mice as indicated by increased level of creatinine (Cr) and blood urea nitrogen (BUN) (Fig. 2O and P). Interestingly, post treatment with PDX facilitated the recovery of function in both liver and kidney (Fig. 2M–P).

### PDX reduced bacterial load and recruited macrophage to peritoneum in septic mice

**Figure 3:**
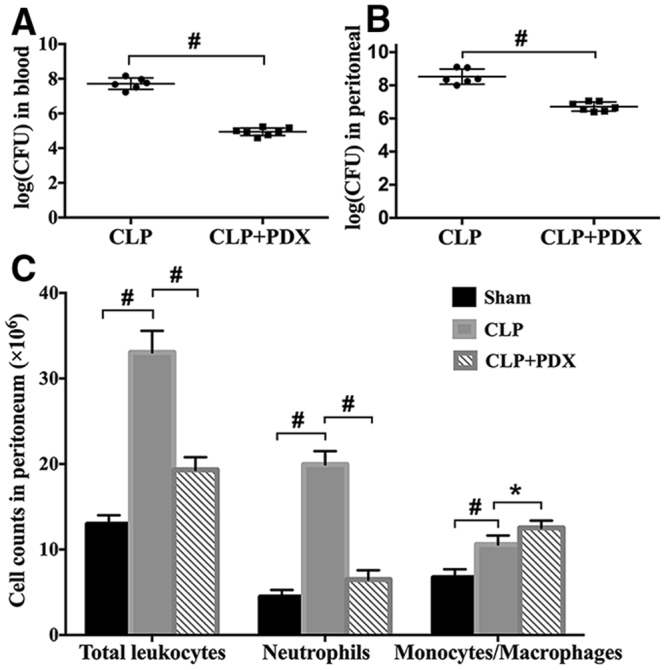
PDX enhanced bacterial clearance and increased the number of macrophage in peritoneum of CLP mice. Bacterial load in both of blood (**A**) and peritoneal lavage fluid (**B**) were decreased by PDX treatment in septic mice. Leukocytes including neutrophils, monocytes and macrophages, infiltrated to peritoneum in response to sepsis (**C**). PDX inhibited the neutrophils infiltration and promoted macrophages migrate to peritoneal cavity of CLP mice (**C**). Data are means ± SEM (n = 6). *P < 0.05, ^**#**^ *P* < 0.01.

Bacterial CFU counts from blood and PLF were collected 24 h after CLP. It was observed that bacterial CFU counts significantly increased in blood and PBL in septic mice (Fig. 3A). Both blood and peritoneum bacterial burden were lowered in mice treated with PDX (Fig. 3B). Next, Giemsa staining was applied to assess the amount of differential cell in peritoeum under sepsis. The amount of infiltrated total leukocytes (especially PMNs) in the peritoneal cavity was increased in septic mice as compared with sham group (Fig. 3C). PDX suppressed neutrophil recruitment in the peritoneum, whereas the number of monocytes and macrophages was increased significantly (Fig. 3D). As macrophages play an important role in the bacteria clearance, these results may suggest that the reduction in bacterial burden is associated with the increased amount of macrophages.

### PDX attenuated inflammatory response in septic mice

**Figure 4:**
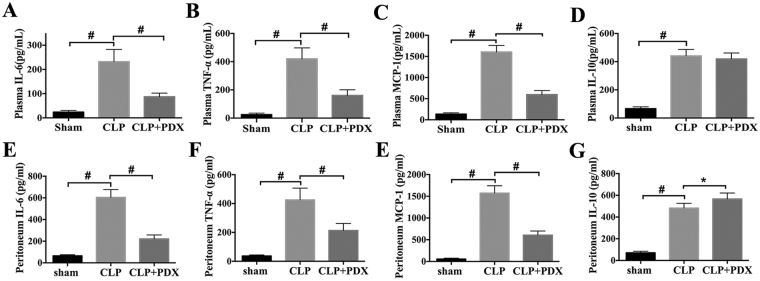
PDX attenuated inflammatory response locally and systemically in sepsis. Plasma and peritoneal lavage fluid (PLF) were harvested 24 hours after CLP. Pro-inflammatory cytokines TNF-a (**A**,**E**), IL-6 (**B**,**F**) and MCP-1 (**C**,**G**) and anti-inflammatory cytokine IL-10 (**D**,**I**) in plasma and PLF were performed by ELISA analysis. Data are presented as mean ± SEM (n = 6). ^**#**^ *P* < 0.01.

To assess the effect of PDX on inflammatory response following CLP, the concentration of cytokines in plasma (systemically) and peritoneum (locally) were detected 24 h after surgery. The concentration of inflammatory cytokines (TNF-a, IL-6, MCP-1 and IL-10) were dramatically increased in plasma and peritoneum in mice under sepsis (Fig. 4A–H). However, PDX reduced pro-inflammatory cytokines (TNF-a, IL-6 and MCP-1) production in both plasma and peritoneum in septic mice (Fig. 4A–C and E–G). In addition, PDX showed no influence on the release of anti-inflammatory cytokine IL-10 in plasma but increased its concentration in PLF from CLP mice (Fig. 4H).

### PDX enhanced phagocytic capability of PMs in mice

**Figure 5:**
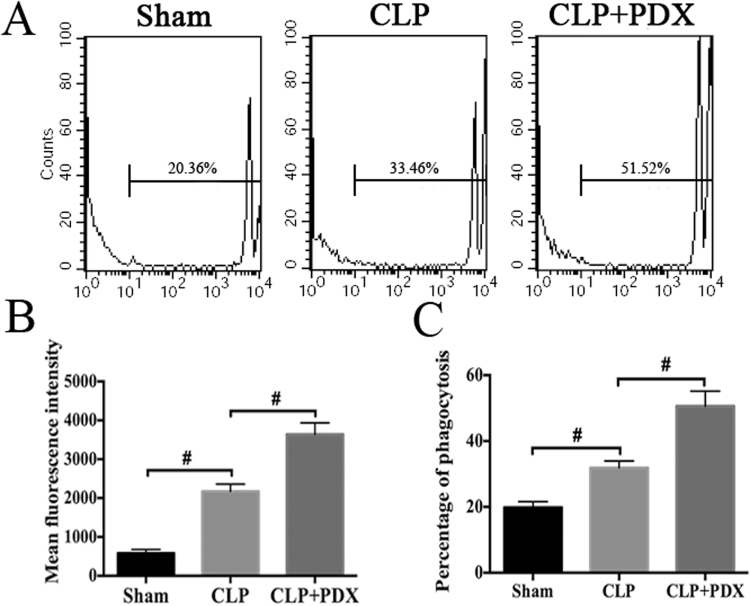
PDX improved phagocytic capability of peritoneal macrophage from septic mice. Peritoneal macrophages were collected 24 h after CLP and incubated with carboxylate-modified microspheres for 1 h and the samples were analyzed by flow cytometry. (**A**) Representative image for each group. The mean fluorescence intensity (**B**) and the percentage of phagocytosis (**C**) were quantified with flow cytometry analysis. Data are presented as mean ± SEM (n = 6). ^**#**^ *P* < 0.01.

We evaluated the effects of PDX on phagocytic capability of PMs by flow cytometry analysis (Fig. 5A). PDX administration increased the mean fluorescence intensity (Fig. 5B) and the percentage of phagocytosis (Fig. 5C) of fluorescent beads as compared with CLP mice.

### PDX promoted M2 polarization in septic mice

**Figure 6:**
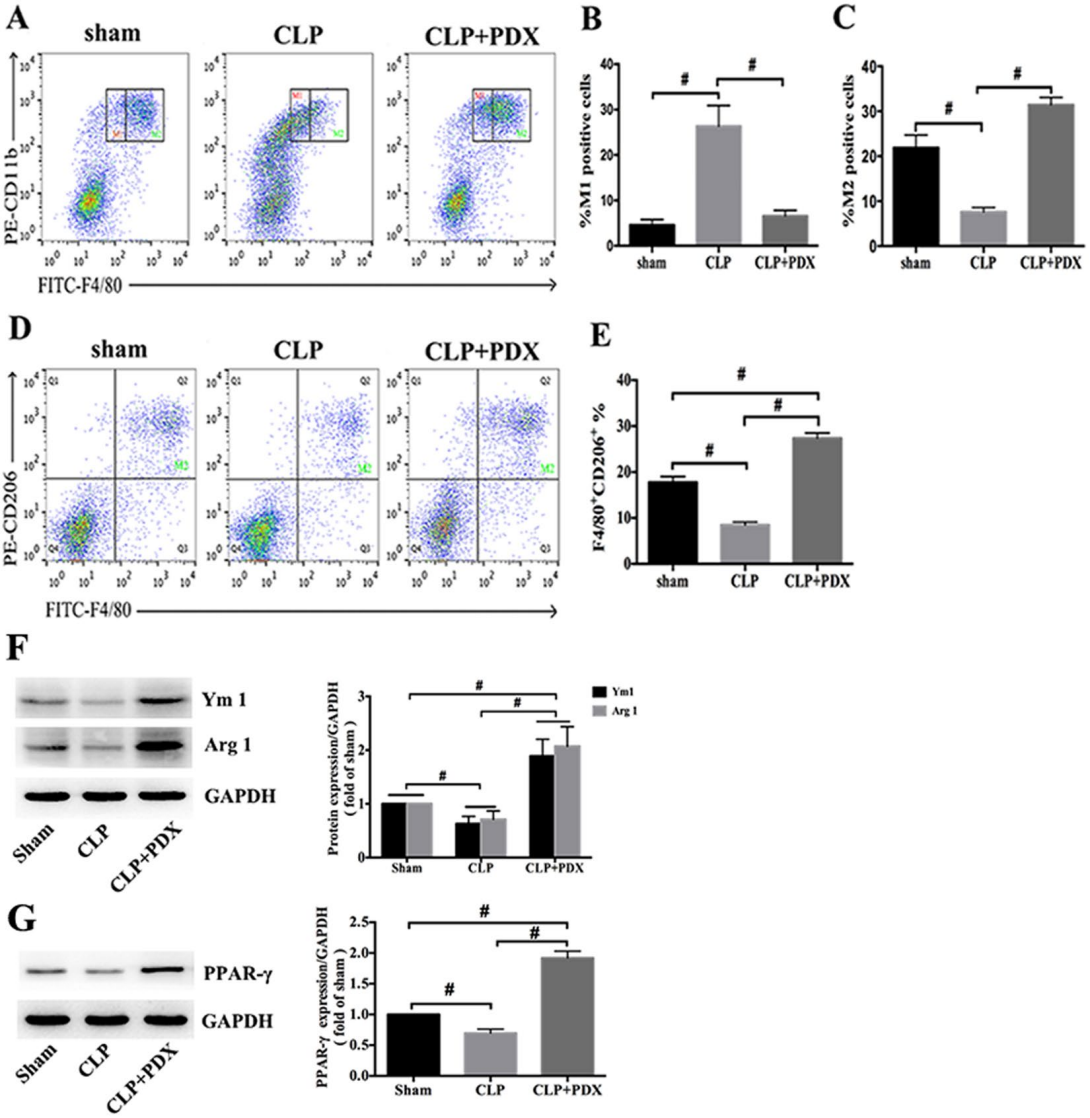
PDX enhanced M2 polarization from peritoneal cavity of septic mice. Peritoneal macrophages were harvested and detected by flow cytometry 24 h after CLP. M2 macrophages (F4/80^hi^CD11b^hi^) were reduced sharply in CLP group when compared with sham group (**A**,**C**). And M1 macrophages (F4/80^int^CD11b^hi^) were increased obviously in CLP mice (**A**,**B**). PDX administration significantly increased M2 macrophages (F4/8^+^CD206^+^) in peritoneum of septic mice (**A**–**E**). The protein levels of Arg1, Ym1 (**F**) and PPAR-γ (**G**) were measured by western blot. Quantification for protein expression was normalized by GAPDH. Data represented the mean ± SEM (n = 6). *P < 0.05, ^**#**^ *P* < 0.01.

To investigate the effect of PDX on macrophage polarization *in vivo*, PMs were obtained and assessed by flow cytometry. Reserchers suggest that relative expression of flow cytometry markers F4/80 and CD11b is able to distinguish subtypes of macrophages, F4/80^int^CD11b^hi^ represents M1 macrophages and F4/80^hi^CD11b^hi^ represents M2 macrophages^22, 23^. However, as this approach may not fully demonstrate the heterogeneity of this population, so we used F4/80^+^ CD206^+^ to indentify M2 macrophages. The percentage of M2 macrophage decreased in CLP mice as compared with mice from sham group (Fig. 6A,C,D). Correspondingly, the number of M1 macrophages increased significantly 24h after CLP (Fig. 6A and B). And PDX treatment significantly increased the proportion of M2 macrophages in septic mice (Fig. 6A–E).

In addition, to confirm the effect of PDX on macrophage polarization, a certain number of PMs (1 × 10^6^) from these three groups were collected 24 h after CLP. M2 macrophage marker (Ym1, Arg1) and its transcriptional regulator (PPAR-γ) were analysed. As shown in Fig. 6F and G, PDX up-regulated the expression of Ym1, Arg1 and PPAR-γ as compared with the CLP group.

### PDX enhanced M2 macrophage polarization in RAW264.7 cells

**Figure 7:**
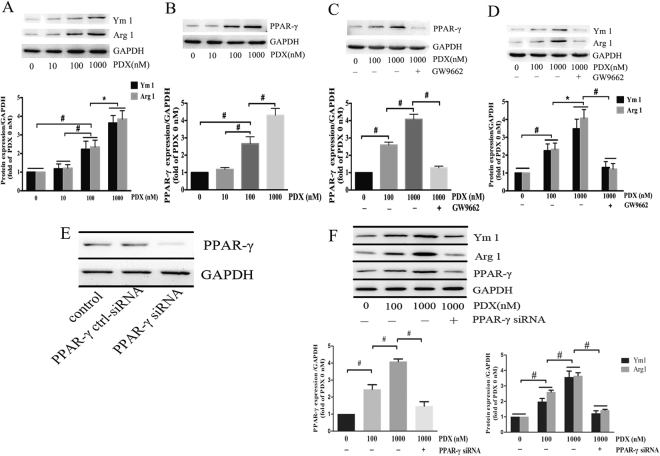
PDX up-regulated the expression of M2 markers in RAW264.7 cells. RAW264.7 cells were stimulated by vehicle (0.03% ethanol) or different concentrations of PDX (10 nM, 100 nM or 1000 nM) for 24 h in the absence or presence of GW9662 (1 µM). The expression of Arg1, Ym1 and PPAR-γ was analyzed by Western blot (**A**,**B**). And the effect of PDX (1000 nM) treatment was blocked by a PPAR-γ antagonist GW9662 (**C**,**D**). The effect of siRNA against PPAR-γ on the expression of PPAR-γ, Ym1 and Arg1 was also assessed by Western blot (**E**,**F**). Quantification for protein expression was normalized by GAPDH. Data represented the mean ± SEM from three independent experiments. *P < 0.05, ^**#**^ *P* < 0.01.

To explore the underlying mechanism on how PDX promote macrophage M2 polarization, RAW264.7 cells were cultured and treated with vehicle (0.03% ethanol) or different concentration of PDX (10 nM, 100 nM and 1000 nM) for 24 hours. The results demonstrated that PDX challenge enhanced the expression of Ym1, Arg1 and PPAR-γ in RAW264.7 cells in a dose-dependent manner (Fig. 7A and B). In order to identify the function of PPAR-γ in macrophage activation induced by PDX, GW9662 (a PPAR-γ antagonist) was applied 30 minutes prior to PDX and the effect of PDX was abrogated by GW9662 (Fig. 7C and D). Furthermore, the effect of PPAR-γ knockdown using siRNA on PDX-induced macrophage polarization was also tested. As shown in Fig. 7E, transfection with siRNA against PPAR-γ down-regulated the expression of PPAR-γ. Surprisingly, PPAR-γ siRNA decreased the expression of Ym1 and Arg1 induced by PDX (Fig. 7F).

**Figure 8:**
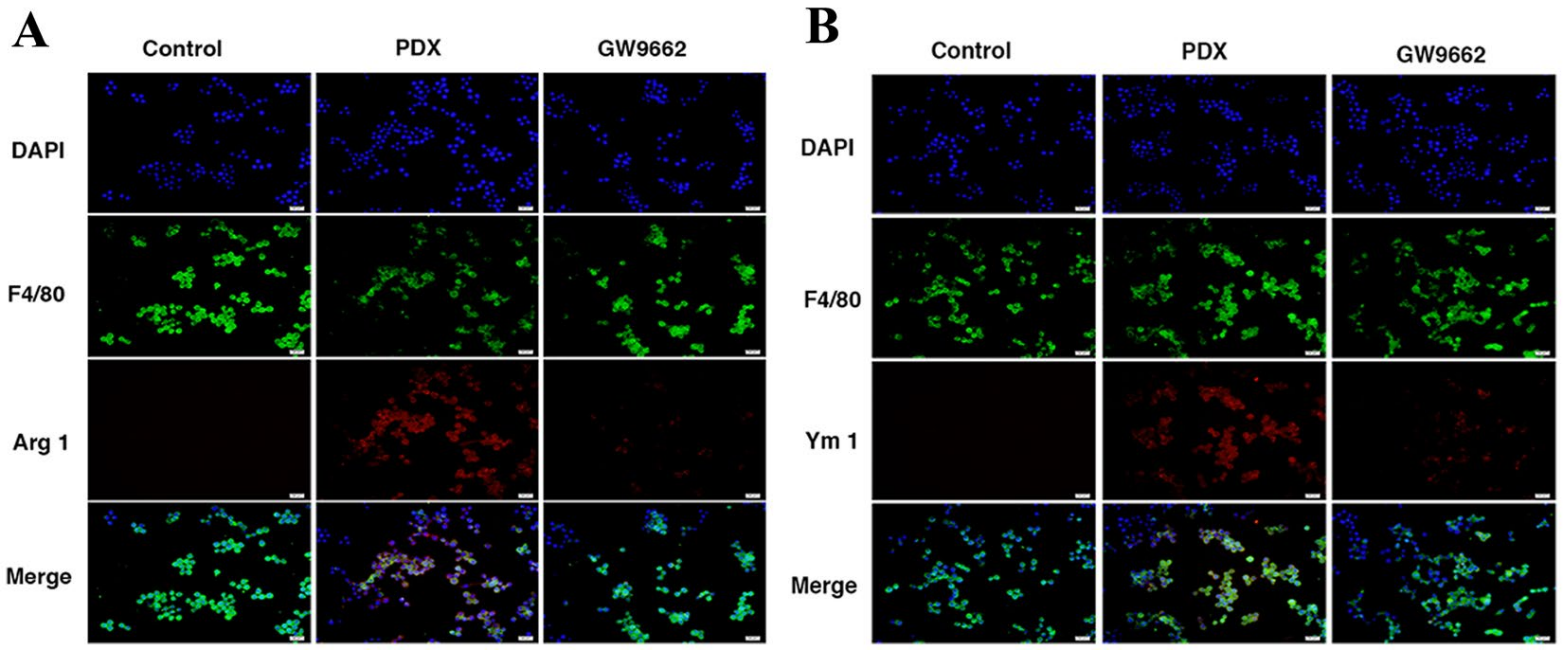
PDX induced M2 polarization *in vitro.* RAW264.7 cells were challenged with vehicle or PDX (1000 nM) in the absence or presence of GW9662 (1 µM) for 24 hours. Macrophage marker F4/80 (pan marker, green) and M2 phenotype makers (Arg1 and Ym1) (red) were measured by immunofluorescence staining (**A**,**B**). Nuclei were stained with DAPI (blue).

Immunofluorescence staining was also performed to verify the effect of PDX on Arg1 and Ym1. As shown in Fig. 8A and B, the expressions of Arg1 and Ym1 were upregulated following PDX (100 nM or 1000 nM) challenge and the effect of PDX (1000 nM) was abrogated by GW9662.

## Discussion

Uncontrolled inflammation, dysregulated immunization and inappropriate accumulation of leukocytes are key pathophysiological characteristics of sepsis^24^. The innate immune cells such as neutrophils, monocytes, and macrophages are recruited to the infection site and then release inflammatory cytokines, resulting in multiple organ dysfunction^25, 26^. The distinct function and remarkable plasticity of macrophages makes it a potential target in treating inflammatory diseases including sepsis^27, 28^. More recently, a series of studies have suggested that pro-resolving mediators (protectins, resolvins and maresins), which are endogenously generated from ω-3 fatty acid, is capable of stopping inflammation signals and promoting the resolution of inflammation in different ways^4, 29, 30^. Particularly, protectins can boost the resolution of inflammation and contribute to restitute host homeostasis^11, 31^. The stereostructure and potent anti-inflammation actions of protectin D1 (PD1, also named as neuroprotectin D1) had been thoroughly investigated^4, 32^. PD1 promoted the resolution of inflammation by decreasing leukocyte infiltration, enhancing macrophage phagocytosis and migrating phagocytes from inflammation site to lymphatic system following efferocytosis^4, 11, 31^. However, as an isomer of PD1, little is known about the exact actions of PDX^9^. PDX is a newly identified di-oxygenated derivatives from DHA, it can be produced by human neutrophils incubated with DHA and can be found together with PD1 in inflammatory exudates^12^. PDX was shown to inhibit platelet aggregation and neutrophil activation by blocking cyclooxygenase-1 (COX-1) and COX-2 as well as antagonizing TxA2 receptor^8, 10^. In addition to its isomer PD1, PDX^32^ (mistaken as PD1 throughout the paper) inhibited the replication of influenza virus and conferred protection against the infectious disease. PDX also stimulated the activation of AMP-activated protein kinase (AMPK) and alleviated insulin resistance in type 2 diabetes by selectively induced the skeletal muscle to release the prototypic myokine IL-6^11^. In the current study, we identified the efficacy of PDX in improving survival rate in a mouse model of sepsis, resolving inflammation, and modulating the peritoneal macrophage phenotype change.

Our data demonstrated that administration of PDX (300 ng or 1000 ng) 1 h after surgery increased 8-day survival in CLP mice. Although PDX 1000 ng treatment displayed slightly higher survival rate than PDX 300 ng in CLP mice, there is no significant difference between the two groups. Therefore, PDX 300 ng was used for the following experiments. As sepsis is the principal cause of multiple organ dysfunction^1^. Here, our study showed that PDX protected mice from sepsis induced organ injury (lung, liver, and kidney) and therefore improved the overall survival rate. Consistent with other DHA-derived mediators, PDX suppressed neutrophils influx to peritoneum and increased the amount of macrophages in peritoneum of septic mice^4, 33^. Moreover, it has been proved that PDX could prevent neutrophil infiltration in a mouse model of peritonitis by 20–25% after administration with a dose of 1 ng/mouse^12^. It is common knowledge that macrophages play a critical role in immune responses. Monocytes and macrophages from infection sites and blood could convert DHA to pro-resolving lipid mediators such as protectins, maresins, and resolvins^34^. Macrophages also produce pro-resolving lipid mediators, and anti-inflammatory cytokine IL-10^16^. PDX enhanced the clearance of bacterial load both of blood and PLF from CLP mice. Many studies indicated that peritoneal macrophages would migrate to local sites of infection, engulf the apoptotic neutrophils and bacteria, thereby contributing to the resoluton of acute and chronic inflammation^34, 35^. As anticipated, PDX treatment enhanced the phagocytosis activity of peritoneal macrophages in CLP mice, indicated by increased number of swollowed fluorescent beads per cell and number of macrophages containing fluorescent beads. It must be emphasized that DHA and its derivants could enhance phagocytosis activity of macrophages, the primary function of M2 macrophages, which is an essential part in the process of inflammation resolution^4, 31, 36^. Our findings support the hypothesis that PDX reduced bacterial load by increasing the recruitment of macrophage and enhancing phagocytosis of bacteria.

PDX reduced the production of pro-inflammatory cytokines, including IL-6, TNF-α and MCP-1 locally and systemically of septic mice. In addition, as an anti-inflammatory cytokine, IL-10 was increased in PLF by PDX in septic mice^37, 38^. Importantly, IL-6, TNF-α and MCP-1 are classical markers of M1 macrophages, and IL-10 is a classical marker of M2 macrophages^39^. These findings suggested that PDX may protect organs by suppressing pro-inflammatory responses and activating macrophages polarization in sepsis. To gain a better insight into the effects of PDX on macrophages, PMs were isolated from mice 24 h after CLP. Surprisingly, our results showed that PDX could promote peritoneal macrophage phenotype change during sepsis in mice. Firstly, the markers (F4/80, CD11b, and CD206) of peritoneal macrophages were shifted from M2 to M1 phenotype after CLP, and PDX treatment changed macrophage phenotype from M1 to M2 in septic mice. Secondly, the expression of functional markers (Ym1 and Arg1) of M2 macrophages was down-regulated in CLP mice as compared with mice in sham group, and PDX administration up-regulated the expression of M2 markers. Notably, as a transcription factor that is able to activate macrophages from different tissues, the expression of PPAR-γ was up-regulated parallel to Arg1 and Ym1^40^. Many investigators have proved that M2 macrophages maintain the homeostasis in peritoneum of mice^41, 42^. M2 macrophages also secreted anti-inflammatory cytokines including IL-10 and limited excessive inflammatory response^43^. Collectively, these findings highlight the critical role of PDX in modulating phenotype change of macrophages during sepsis.

In order to confirm the effect of PDX on polorization of macrophage *in vitro*, RAW264.7 cell line was cultured and incubated with different concentration of PDX. It was found that the expression of Arg1, Ym1 and PPAR-γ were markedly up-regulated in RAW264.7 treated with PDX in a dose-dependent manner^16^. It was also reported that the expression of M2 macrophage markers were obviously decreased after being treated with PPAR-γ inhibitor (GW9662)^44^. And the same results were obtained by Western blot analysis and immunofluorescence staining. Moreover, knockdown of PPAR-γ gene expression using siRNA down-regulated the expression of Ym1 and Arg1 induced by PDX. Therefore, we reasoned that PDX could promote M2 polarization through PPAR-γ dependent signalling pathway.

In conclusion, PDX confers protection against sepsis in mice by enhancing phagocytosis of macrophages, decreasing inflammatory response and facilitating M2 polarization of peritoneal macrophages.

## Methods

### Reagents and antibodies

Mouse RAW264.7 macrophages were obtained from American Type Culture Collection (Manassas, VA, USA) and maintained in Dulbecco’s modified Eagle’s medium (DMEM) (Hyclone, Logan, UT, USA) containing 10% fetal bovine serum (Gibco, Life Technologies). PDX was purchased from Cayman (Cayman Chemical, Ann Arbor, MI, USA). IL-6, TNF-α, MCP-1 and IL-10 (enzyme-linked immunosorbent assay, ELISA) kits were obtained from RayBiotech (RayBiotech Inc., Norcross, GA, USA). Carboxylate-modified yellowgreen (YG) microspheres were purchased from Invitrogen (Thermo Fisher scientific, Waltham, MA, USA). FITC-anti-F4/80, PE-anti-CD11b and PE-anti-CD206 were obtained from eBioscience (eBioscience, San Diego, CA, USA). Anti-Arg1 antibody was purchased from Abcam (Abcam, Cambridge, MA, USA). Anti-Ym1 antibody was purchased from Stemcell Technologies (Stemcell Technologies, Vancouver, Canada). Anti-PPAR-γ and anti-GAPDH antibodies were obtained from Santa Cruz Biotechnology (Santa Cruz, CA, USA).

### Animals

6–8 weeks old male C57BL/6 mice weighing 20–25 g, were obtained from the Animal Center of Wuhan University (Wuhan, China). The mice were housed under a 12-h light-dark cycle, with food and water provided ad libitum. All experimental procedures involving animals were performed in accordance with the NIH guidelines and approved by the Animal Committee of Tongji Medical College (Wuhan, China).

### Cecal ligation and puncture model

CLP model was performed as described previously^45^. Briefly, mice were anesthetized with isoflurane inhalation. The cecum was exposed after a longitudinal skin midline incision was made in disinfected abdomen. Ligate the cecum at the desired position for mid-grade sepsis. Perforate the cecum by through and through puncture with a 20-gauge needle and extrude a droplet of feces from holes to ensure patency. The cecum was returned to the abdomen, which was closed in two layers. For sham control, the cecum was exposed but not ligated or punctured, and then placed back into the peritoneal cavity. 1 ml of prewarmed (37 °C) saline was injected subcutaneously to resuscitate animal after surgery. CLP mice were administered either PDX (Cayman Chemical, Ann Arbor, MI, USA) or vehicle (0.1 ml saline) intraperitoneally 1 h after surgery.

### Survival analysis and histological assessment

Mice underwent CLP with or without PDX administration were observed every 24 h for 8 days for survival analysis. Parallel experiment was conducted as follows for histological assessment. Mice were sacrificed 24 h after the procedures, then the lung, liver and kidney were removed immediately after exsanguination. Sections were stained with hematoxylin-eosin after these organs were embedded in paraffin. Organ injury scores were performed by an investigator blinded to the study according to the published criteria^46–48^.

### Measurement of blood biochemistry

Whole blood from mice was collected 24 h after CLP. Then the concentration of alanine aminotranferase (ALT), aspartate amniotransferase (AST), creatinine (Cr) and blood urea nitrogen (BUN) in blood was determined by using Olympus AU400 automated chemistry analyzer (Olympus, Tokoyo, Japan).

### Bacterial load and differential leukocyte counts

Blood and peritoneal lavage fluid (PLF) were collected 24 h after CLP. Then blood and PLF were spread on tryptic soy blood agar plates after serially diluted with phosphate buffered saline (PBS). Plates were incubated 24 h in aerobic conditions at 37 °C, then the colony-forming units (CFU) was counted. Total PLF cells were assessed by using a haematocytometer. Briefly, cells in PLF were spun onto microscope slides at 1000 rpm for 5 mins using cytospins (Thermo Fisher Scientific, Waltham, MA, USA). And then differential cell counts were determined under a light microscope after stained with Giemsa.

### Cytokine detection

The IL-6, TNF-α, MCP-1 and IL-10 levels in the plasma or PLF were measured by ELISA kits (RayBiotech, Inc. Norcross, GA, USA) according to the manufacturer’s protocol.

### Peritoneal macrophage isolation

Peritoneal macrophages (PMs) were obtained from mice as described previously^49^. Briefly, the peritoneal cavity was lavaged with 20 ml cold PBS, and the PLF was centrifuged at 250 g for 5 min. Cells were resuspended in RPMI 1640 medium (Hyclone) containing 10% FBS and incubated in a humidified incubator with 5% CO2 at 37 °C for 2 hours. And then non-adherent cells were removed by gentle washing with PBS. PMs were used for following experiments immediately after isolation.

### Phagocytosis assay

PMs were seeded in 6-well plate at a density of 5 × 10^5^ cells/well and incubated with 2 μl carboxylate-modified yellowgreen (YG) microspheres (505/515 nm wavelength, diameter 2 µm; Invitrogen) for 1 h at 37 °C in dark. Cells were analyzed by FACSCalibur flow cytometer (BD Biosciences, San Jose, CA, USA) equipped with Argon laser after washing with cold PBS. Mean fluorescence intensity (MFI) and the percentage of phagocytosis were evaluated by counting the cells containing microspheres and detecting the wavelength emission of the fluorescent microspheres.

### Flow cytometry analysis

PMs were harvested 24 hours after CLP and washed with cold PBS twice, and then resuspended in FACS buffer. Collective cells were incubated with FITC-anti-F4/80, PE-anti-CD11b, or PE anti-CD206 for 30 min at 4 °C in darkness, respectively. Then cells washed twice by centrifugation with FACS buffer, and resuspended in 200 µL PBS analyzed by FACSCalibur using the Cell Quest software. Appropriate isotype controls were performed in all cases. FlowJo software (Tree Star, Ashland, OR, USA) was used to analyze the data.

### Transfection of siRNA

The siRNA against PPAR-γ and an irrelevant scramble siRNA as a control were purchased from Santa Cruz Biotechnology (Santa Cruz, California, USA). RAW264.7 cells at the density of 5 × 10^5^ cells/well were incubated in antibiotics free Opti-MEM Reduced Serum Medium (Invitrogen) with PPAR-γ siRNA or control siRNA in the presence of appropriate plasmids using Lipofectamine 2000 (Invitrogen). Then the medium was changed to fresh DMEM after 4 h incubation, and Western blot analysis was performed to assess the effectiveness of siRNA against PPAR-γ. RAW264.7 cells were transfected with siRNA 48 h prior to PDX challenge in the following experiments.

### Western blot analysis

PMs and RAW264.7 cells were washed with cold PBS and lysed with a buffer containing cocktail (a protease inhibitor cocktail) for 15 min. After centrifuged at 12000 rpm for 15 minutes at 4 °C, the protein concentration was determined by BCA Protein Assay kit (Thermo, Rockford, IL). Equal amounts of protein were separated by 10% SDS-PAGE and then transferred to a PVDF membrane (Millipore Corp., Billerica, MA). The membranes were blocked with 5% non-fat milk for 1 h and probed with indicated primary antibodies at 4 °C overnight. On the next day, the membranes were incubated with second antibodies for 1 h at room temperature following three times wash in Tris-bufftered saline with Tween 20. After washing, the membranes were incubated with chemiluminescence reagents (Beyotime Institute of Biotechnology, Shanghai, China) and detected using UVP imaging system (Upland, CA, USA). The obtained images were analyzed by Image J software. Following antibodies were used in the study: Arg1, Ym1, PPAR-γ and GAPDH.

### Immunofluorescence staining analysis

Cultured cells were fixed with 4% paraformaldehyde in PBS for 1 h and then permeabilized with 0.1% Triton X-100 (Sigma, St. Louis, MO, USA) in PBS for 10 min. After blocked with 3% BSA in PBS for one hour, cells were incubated with primary antibodies (F4/80, Arg1, or Ym1, diluted with 1% FBS in PBS). After rinsing, cells were incubated with indicated secondary antibodies for 1 h and counterstained with DAPI to identify cell nuclei. The cells were mounted in antifade mounting medium (Beyotime, Shanghai, China) after rinsed with PBS, and the cell staining was photographed under an immunofluorescence microscope (Olympus, Tokyo, Japan).

### Statistical analysis

Survival rate in different groups were analyzed by a Kaplan-Meier survival curves and log-rank statistics. Data are presented as mean ± SEM and were analyzed by Graphpad Prism (version 6.0, USA). Multiple comparisons were analyzed using One-way ANOVA followed by least significant difference *post hoc* test. *P* < 0.05 was accepted as statistically significant.
